# Being different during treatment: a qualitative study investigating patients’ experiences of treatments for missing maxillary lateral incisors

**DOI:** 10.2340/aos.v83.42315

**Published:** 2024-11-12

**Authors:** Cecilia Hedmo, Rune Lindsten, Eva Josefsson, Aimée Ekman

**Affiliations:** a Department of Orthodontics, The Institute for Postgraduate Dental Education, Jönköping, Sweden; b School of Health and Welfare, Jönköping University, Jönköping, Sweden

**Keywords:** Aplasia, lateral incisors, treatment experience, qualitative methods, patient perspective

## Abstract

**Introduction and objective:**

Agenesis of one or more teeth is common among patients who are referred for orthodontic treatment. The most common treatments are orthodontic space closure (SC) and implant replacement (IR), which are widely studied, but the experiences of patients receiving these treatments have received little attention. The aim of this qualitative study is to explore how treatments to address missing maxillary lateral incisors (MMLIs) are experienced by individuals who are treated using either orthodontic SC or IR.

**Materials and methods:**

This study is conducted in Sweden and based on semi-structured interviews with 13 individuals who have completed treatment, either orthodontic SC (*n* = 7) or IR (*n* = 6), to address the lack of one or two maxillary lateral incisors. Data were analysed in accordance with the grounded theory approach.

**Results:**

Findings were classified into the main category of *being different during treatment* and into three associated sub-categories. The first category, that is *being different due to missing teeth,* refers to when a person experiences being different because of the anterior spacing The second category, that is *being different due to fixed appliance*, refers to when the appliance itself makes a person different. The two first categories exemplify being different in terms of appearance. The third identified category, that is *being different due to treatment appointments,* refers to the need to spend time differently because of having appointments at the clinic for treatment.

**Conclusion:**

Patients MMLIs consider their treatment to start at the time of diagnosis. They experience feelings of being different irrespective of whether the type of treatment is orthodontic SC or IR. The experience of being different differs in timing and causes depending on the treatment method.

## Introduction

Agenesis of one or more teeth is common among patients who are referred for orthodontic treatment. The prevalence of agenesis ranges between 2.5% and 6.9% [1], and the teeth most often affected are the maxillary and mandibulary second premolars and the maxillary lateral incisors [2]. Several studies have reported the prevalence of agenesis of the maxillary lateral incisor as ranging from 1.5% to 2.0% [1, 3].

When choosing a treatment to solve the problem of a missing tooth, it is common practice to choose the least invasive treatment with the best long-term prognosis. For many patients, several treatment alternatives are possible, and the three major treatment alternatives are as follows: space closure (SC), prosthetic replacement, and auto-transplantation. Among these options, the two treatments that are most commonly used are orthodontic SC and redistribution of space with orthodontic appliances to enable prosthetic replacement of the missing tooth [4]. Prosthetic replacement of a missing maxillary lateral incisor (MMLI) may be performed with conventional tooth supported bridges or resin bonded bridges. However, osseo-integrated implants and implant-supported crowns are considered to be the treatment of choice in cases where the conditions are sufficiently favourable for their use [5, 6]. The different treatments are more or less suitable among individuals depending on age of the patient and type of malocclusions [4, 7].

Left untreated, MMLI is known to have a negative impact on people’s oralhealth-related quality of life (OHRQoL) [8]. However, in what ways and to what extent missing teeth influence OHRQoL is not yet fully understood [9]; even less is understood about patients’ quality of life (QoL) during treatment due to missing maxillary laterals. Major focus has so far been on studying the effect of appliance on QoL, OHRQoL in patients during and after completed treatment, and the treatment outcome. The majority of the studies published within the field of orthodontics that have investigated patient experiences of treatment and treatment outcomes are quantitative and based on questionnaires [10]. Aesthetic outcomes in patients treated because of MMLIs have been studied by many researchers [11–14] and is considered to be equal according to laymen and professionals. Mohammed et al. [15] found in their qualitative meta-synthesis that orthodontic treatment positively influenced patients’ self-esteem, social interactions, and their aesthetics. Challenging aspects of the orthodontic treatment that have been reported are discomfort and pain [16], changes in diet [17, 18], and difficulties in maintaining good oral hygiene [17].

A smile with a large display of natural, white, and symmetrical teeth without spacing is considered the desirable norm [19, 20]. Research has shown that individuals with an attractive smile are perceived to be more intellectual and to have higher social abilities based only on their dental aesthetics [21, 22]. Orthodontic treatment is offered and performed in order to adjust dental function and aesthetics for individuals with dental deviations [23].

Orthodontic treatment with fixed appliances is performed for 20 months on average [24], but in some cases the duration of this treatment is extended [25]. Hence, an orthodontic treatment implies a long-term relationship between patient and caregiver, and it requires that the caregiver keeps up-to-date with the caretakers’ feelings regarding the treatment [26]. Patients’ experiences of treatment have not yet been extensively researched. The current lack of knowledge on how treatment due to MMLI is experienced by patients treated with either orthodontic SC or implant replacement (IR) highlights the significance of this study.

The aim of this qualitative study is to explore how treatment is experienced by individuals who are treated using either orthodontic SC or IR to address MMLIs.

## Methods

Data collection and analysis were performed in accordance with the grounded theory approach, as outlined by Kathy Charmaz [27]. Grounded theory is an inductive, comparative, iterative, and interactive method used to develop a conceptual understanding of the collected data [27]. The research process for this study started with a broad interest in individuals’ experiences from two different treatments addressing MMLI, and it resulted in an analysis of patients’ experiences of being different while undergoing treatment for MMLI.

### Settings

This study was conducted in Sweden. When a malocclusion is detected within a general dental care service in Sweden, the general dental practitioner is responsible for consulting a specialist in orthodontics. After this consultation, the patient is offered treatment dependent on the status, occlusion, and subjective need for treatment. If the patient accepts the offered treatment, the general practitioner sends a referral to the orthodontist. The referral is categorised depending on the planned treatment; thus, the course of treatment and the expenditure of time from referral to examination at the orthodontist differs between patients. If the patient will be having pre-prosthetic orthodontic treatment and subsequent IR of the missing tooth or teeth, then this is planned in cooperation with a specialist in prosthodontics.

In Sweden, Dental Health Services are free of charge until the age of 23 years. Orthodontic care aims to treat malocclusions to avoid tissue damage, functional problems, and psychosocial problems. In a subsidised system such as that in Sweden, priorities need to be made to make sure that the right care is provided to the right people. Hence, not every individual who wishes to alter the aesthetics of their dentition is offered orthodontic treatment free of charge.

### Purposive sample

A purposive sample was used to identify potential study participants who were treated because of missing one or two maxillary lateral incisors. A search for individuals to interview was performed within the dental records of a department of orthodontics in a town in southern Sweden. Individuals with oligodontia or cleft lip and palate were excluded from this study at this stage, as their treatment differs from the two types of treatments we were interested in; it is often longer, and not all of the treatment is performed at the orthodontic clinic. No other malocclusions or dental features resulted in exclusion. A total of 15 individuals were contacted by phone (by CH) and informed about the study, 13 of whom decided to participate.

### Study group

The study group consisted of 13 individuals who had completed orthodontic treatment for at least 6 months but not more than 18 months prior to their participation in this study (see Table 1). Seven of the participants had been treated with orthodontic SC, and six had been treated with orthodontic redistribution of space and IR. The interviewer had not been part of any of the treatments, thus had no relation to the interviewees. Participants’ ages ranged from 18 to 25 years except for one participant, who entered treatment later in life (after 30) and was 35 years old when the interview was conducted. Implant replacements were performed when the patients were 20 years or older. MMLIs are more common in women than in men, and this gender distribution is reflected in the study group, which was composed of 10 females and 3 males. All participants lived in the southern parts of Sweden and had Swedish as their native language.

**Table 1:** Presentation of interviewees

Group	*N*	Age	Sex		Agenesis		Time (months)
		(years)	Male	Female	Unilateral	Bilateral	since treatment
IR	6	22–25 (35)	2	4	3	3	6–17
SC	7	18–21	1	6	3	4	6–14
Total	13	18–25 (35)	3	10	6	7	6–17

Presentation of interviewees treated with either implant replacement (IR) or orthodontic space closure (SC).

### Data collection and analysis

In accordance with the grounded theory approach, data collection and analysis were conducted in an alternating manner [28]. Semi-structured interviews were undertaken in a non-clinical area (*n* = 11) or by using Zoom (©2024 Zoom Video Communications, Inc.) (*n* = 2) by the first author (CH) at a department of orthodontics, and each interview lasted 30–40 minutes. The interviewer used open-ended questions that were designed to cover a wide range of treatment experiences. Participants were encouraged to determine the flow and content of the interviews. Interruption and guiding questions were avoided [27], but invitational follow-up questions were used by the interviewer [29].

All interviews were conducted in Swedish, and they were recorded and transcribed verbatim shortly after the interview by one of the authors (CH). The analytical process included the following steps: initial coding of the empirical material, meaning unit by meaning unit and also in larger segments, such as sentences, paragraphs, and across cases; categorisation, which involved grouping the initial codes into categories based on their similarities and differences; focussed coding, which comprised of more selective and conceptual coding; and conceptualisation, which is a procedure for transforming categories into concepts [27].

The analysis also targeted similarities and differences between the accounts from participants who had undergone either treatment. Categories and data were constantly compared, and data collection continued until theoretical saturation was established, meaning that the researchers had created all of the dimensions and sub-dimensions available from the material [27].

### Ethical considerations

This study was approved by the Swedish Ethical Review Authority, diary number 2020-00301. The participants were informed about the study and gave their consent – both orally and in writing – to participate. Participants were free to withdraw their consent at any time during the research process; none of the participants exercised this right. The interview material, audio, and text from the interviews has only been accessed by the authors. Personal data were managed in accordance with the EU General Data Protection Regulation (GDPR2016/679).

## Results

**Figure 1:**
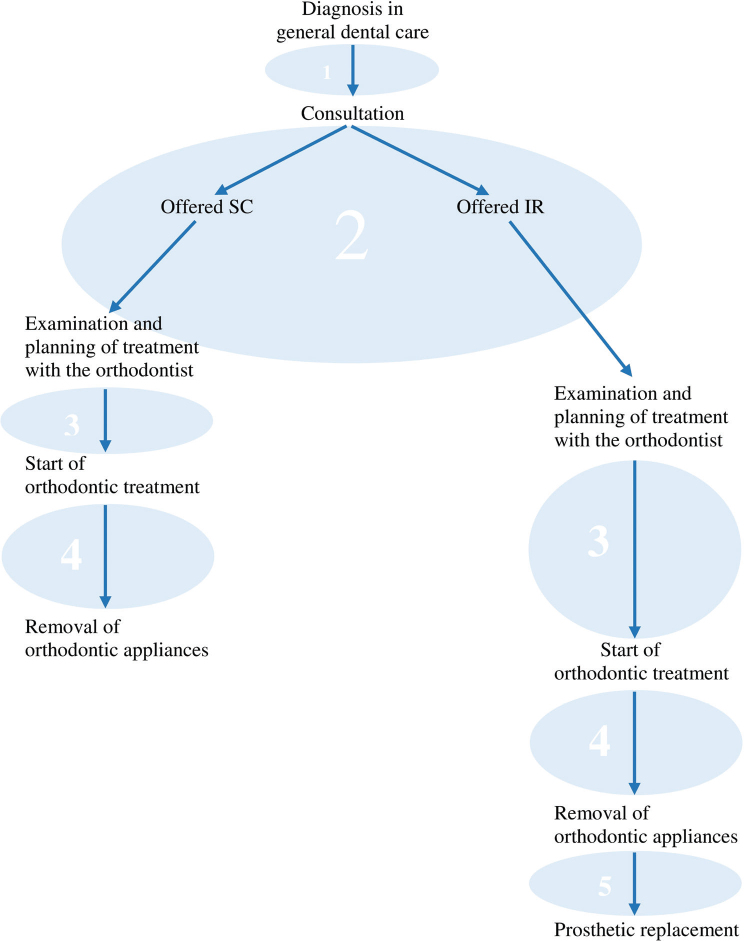
Course of treatment. Treatment course starts when a malocclusion is detected, in this case missing maxillary lateral incisor (MMLI). Following consultation, the patient is offered either orthodontic space closure (SC) or implant replacement (IR). The numbered circles illustrate the different phases of the treatment course, and the different arrow lengths reflect the different lengths of time spent within the different phases.

All participants, except the one who began treatment later in life, told their ‘treatment-story’ starting from the time of diagnosis or from when their dentist informed them that some kind of treatment was necessary or possible, either now or in the future, to address their missing teeth. It became evident that the treatment started at diagnosis according to the interviewees, making the course of treatment prolonged compared to our prior understanding, that resulted in treatment durations that differed from those we had expected. The course of treatment is illustrated in Figure 1, that depicts the different treatment phases, with duration differences illustrated by arrows of different lengths.

The scope of the interviews was broad in the beginning in order to cover a wide range of experiences and beliefs about the targeted treatment methods. A flora of experiences was described, and many factors seemed to influence how the participants experienced their treatment periods. One thing that stood out in the material was the description of being different during treatment. Preliminary categories reflecting the descriptions of being different were developed after eight interviews, with four participants representing each treatment.

During the analysis, questions such as ‘what is this about’, ‘what does this mean’, and ‘how can it be defined’ were asked [30].

Experiences of appliance breakage, difficulties when eating, and pain during treatment were mentioned in all interviews, but mostly in passing and such experiences were described as things that were expected and legitimate given the treatment being received. The things that stood out as significant in the data were the stories of being different during treatment. The analyses presented here reflect this focus and examine the main category *being different during treatment* along with three associated sub-categories.

### Being different during treatment

All of the interviewees expressed the wish for improved aesthetics as a need to fulfil existing social norms and to improve their psychosocial wellbeing as their main reason for treatment, which is in agreement with previous research findings [31–33].

**Figure 2:**
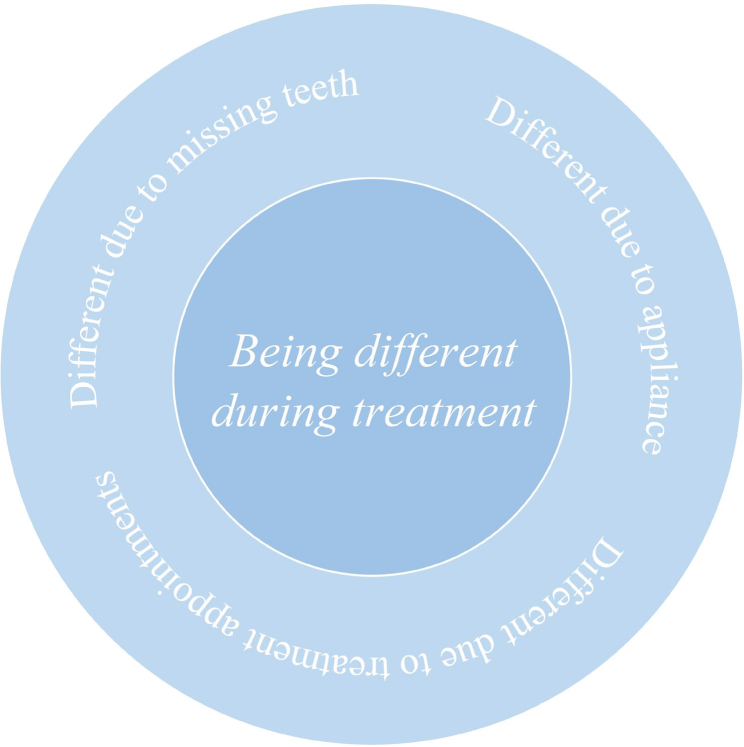
Illustration of main category and subcategories.

Experiences of ‘being different’, as it was expressed in the interviews, consisted of three categories of experiences, which will be described in the following pages and are shown in Figure 2. The first category – *being different due to missing teeth* – refers to when a person experiences being different because of the anterior spacing. The second category – *being different due to a fixed appliance* – refers to when the appliance itself makes a person different. The two first categories exemplify being different in terms of appearance. The third identified category – *being different due to treatment appointments* – refers to the need to spend time differently compared to oneself or peers because of having appointments at the clinic for treatment.

Having experiences of being different can refer to the whole course of treatment or to certain phases within the course of treatment (see Figure 1).

Explanations of being different indicates that a person is deviating from what is considered normal. The analyses performed reflected participants’ descriptions of being different in two ways: being different compared to others and being biographically different. The latter refers to being different compared to how one has been before or what one is normally doing.

### Being different due to missing teeth

Experiences of being different due to missing teeth were expressed independent of treatment method. However, how and when it was expressed depends on treatment. In interviewees treated with SC, being different due to missing teeth was described as being more evident during phase 2, when extractions were performed to enable mesial drifting prior to the fixed appliance.

‘When you start extracting teeth when you are about ten years old I guess it’s kind of common missing teeth in that age. But then you start noticing that they [the friends] get new teeth or so but I didn’t since I didn’t have the succeeding teeth.’

Spacing is part of normal dental development during the period of mixed dentition. What differed for the interviewees was that others had succeeding teeth erupting while they continued to have missing teeth, and this made them different. This spacing decreased when active treatment started with a fixed appliance, which seems to have reduced the inconvenience of missing teeth.

Interviewees treated with IR described themselves as being different due to missing teeth during parts of active treatment, phase 4. Their appearance changed because of space redistribution, which increased the visibility of the missing tooth or teeth. Hence, the treatment in itself increased the difference in appearance compared to peers but also compared to how their teeth looked before. The treatment made it more obvious that there were teeth missing, which was described as troublesome.

‘Initially I had a general spacing. But when starting treatment with braces they [the teeth] changed so that there was two large openings in the front of the mouth. That was the hardest part, that it was so obvious that I was missing teeth.’

The IR-interviewees expressed relief at receiving the temporary prosthesis. Some of them suffered fractures or issues with the temporary prosthesis that resulted in a sudden change in their dental appearance. The interviewees talked about how it made them feel different, ‘it made me feel really odd’, and how their friends reacted to their sudden change in dental appearance when this happened, phase 5.

‘I cried really hard every time it [the tooth] came off because it made every one ask why I didn’t have a tooth there. Because you know it came off now and then. It was like one day the tooth would be there and the next it was gone. Then I had to wait for a week or so before I got it back. After like a month it [the tooth] came off again and so it went on.’

Among the descriptions of being different due to missing teeth, there were also more gripping stories of how the interviewee’s experiences of their dental appearance had affected them and their wellbeing. To some extent, these experiences were woven together with delayed alleviation of dental anxiety. These stories illustrate how much the deviated dental appearance affected the individuals and made them adjust their behaviour.

‘The hardest part about reminiscence is that I have no… [pauses, cries, gathers again] I have no pictures of myself smiling or laughing from when I was younger.’

This was only expressed in interviews by people treated with IR, illustrating that the prolonged period before active treatment affected some of them significantly. Two of the interviewees were affected to such an extent that they became sad and started crying during the interview.

Dissatisfaction with dental aesthetics due to missing teeth were in some cases interpreted as frustration about delayed alleviation of dental anxiety in some IR-interviewees. They expressed expectations of positive effects on their dental appearance because of the appliance.

‘When I was younger I’ve always thought that why cannot I just get those braces now because it doesn’t matter if the treatment will be prolonged. It won’t matter if I must have the braces on for 10 years because braces looks better than this. With braces I can at least smile.’

### Being different due to fixed appliance

The category of being different due to fixed appliance emerged from stories told by all of the interviewees, and refers to when the fixed appliance itself makes a person feel different compared to others.

Most of the interviewees felt that the braces impaired their dental aesthetics, and some even had their confidence diminished by wearing braces.

‘I simply had a lack of confidence. Since none of my friends had braces I didn’t want one either. I just didn’t want to stand out.’

Others made similar statements that they did not want braces even if they chose treatment to improve their dental aesthetics. As the above-stated quote shows, the reason for not wanting braces was that it made them different in a negative way: different in relation to the aesthetic norm but also different compared to their peers who had no braces. However, the interviewees noted that they were not the only ones wearing braces among their peers and friends. Some of the interviewees, mostly SC-interviewees, compared the type of braces they had with their peers who had no appliance or removable appliances. The fixed appliance was described as highly visible and as influencing their appearance in a negative way at all times.

‘You know I felt I got my braces quite early because no one else than me had them [braces]. Others [classmates and friends] hade some sort of removable appliances.’

Even if others might have removable appliances, the SC-interviewees described a sense of belonging and fellowship with other peers and friends having appliances.

‘It’s kind of a community with your friends [that also have braces], as if you have a club where you know what all of you go through. You support each other and you rejoice with each other when you finish [treatment].’

Shared experiences with others having appliances could also to some extent help them to cope with difficulties during treatment and to celebrate when the treatment was completed.

### Being different due to treatment appointments

Being different due to treatment appointments refers to when a person is unable to participate in planned or scheduled activities while having appointments at the dentist for treatment. Having recurring appointments during school hours was noted by teachers and made the interviewees, SC and IR, different from their peers, who mostly went to the dentist around once a year.

‘Well, everybody did not have braces or appointments at the dentist every month.’

In this quote, the person positions themself as being different from other peers in two regards: firstly, for having braces; and secondly, for having appointments at the dentist more often than others did. Including both these aspects in the same sentence indicate that they are both experienced as making this interviewee different compared to peers and/or friends. What differs is that the latter refers to being different because of what one had to do. Being absent from class for the recurring appointments was also described as frustrating because the treatment appointments resulted in missing important school time. To some participants, who were mostly IR-interviewees, it was described as stressful to be absent from school, whereas for others it was no big deal.

‘There are quite many appointments at the dentist and the school doesn’t want you to make them all during school time. So, you try to schedule them before or after school and that result in quite much spare time getting lost. For example, I do horseback riding a lot and the treatment has taken time from that.’

As shown in this quote, there were examples of the need to skip leisure activities, for example horseback riding or soccer practice. The need to skip activities that one would normally do and to spend time differently causes feelings of being different relative to what one is normally doing.

## Discussion

According to our interviewees, the treatment started at the time of diagnosis; that was much earlier than our pre-understanding of when the treatment started, which was at the start of active treatment with fixed appliances. This finding helps us to understand that the interviewees reported treatment as being longer and including more phases than we first anticipated. Aspects addressed in previous studies of orthodontic patients, such as experiences of complications and pain during treatment [10, 16–18, 34–36], were mentioned in all interviews but mostly in passing and while being described as something that was expected and legitimate given the treatment being received.

Independently if the interviewee went through SC or orthodontic redistribution of space and IR, they experienced feelings of being different. Experiences of being different seemed to differ in timing and causes depending on the type of treatment received. If these findings, timing of treatment initiation and feelings of being different, only applies to MMLI-patients or if it is applicable to all patients diagnosed with a malocclusion is not established and for further studies to investigate.

The results of this study make it clear that patients experience being different because of having missing teeth, fixed appliance, and attending treatment appointments. It is notable that the interviewees did not explain the dental norms they deviated from during treatment; these norms were more or less taken for granted, and were made visible through their explanations of how they deviated from them. The experiences of being different that were reported implicitly referred to an aesthetic norm of white and symmetrical teeth without spacing and what participants considered to be normal contact with the dentist.

Being different can be positively loaded, and a choice that one makes to stand out. In contrast, the examples of being different during treatment that were described by the interviewees were unfavourable, and were sometimes even described as problematic. This could be related to the fact that the treatment was carried out during adolescence for all but one of the interviewees. In the adolescence period, major biological, cognitive, and social changes occur in a person that make questions of one’s own identity central [37]. Hence, it is a turbulent time in life for many, where these individuals may feel that a treatment aiming to normalise the dental aesthetics is put on hold. Although the interviewees did not express a desire to achieve perfection, they clearly wanted to improve their aesthetics in so as to fit in with society’s norms. The desire to avoid feeling different is not, in any way, exclusive to the individuals interviewed in this study [10, 23].

Individuals with smiles deviating from the dental norm can be targeted by negative stereotyping, which may affect acceptance by peers [38, 39]. However, such negative prejudices from peers were not described during the interviews.

One advantage reported in the existing literature in favour of SC compared to IR is that SC can start earlier; hence, treatment can be completed during adolescence and the waiting becomes shorter [40]. However, starting treatment earlier results in an individual receiving a fixed appliance earlier than most of their peers; this resulted in feelings of being different due to appliance in some of the SC-interviewees. This finding highlights the importance of including the patient in decision-making. What the profession consider an advantage – an early start to treatment – may not be an evident advantage according to the patient.

When the clinical conditions are in favour of IR of the missing tooth or teeth, the required pre-prosthetic orthodontics and implant treatment is recommended to be delayed for as long as possible [41, 42]; this is to reduce the risks of complications such as discoloured gingiva, visible implants, and infraocclusion of the implant-supported crown [43]. The period of waiting prior to starting active treatment with appliances (which includes phases 2 and 3) resulted in feelings of being different due to missing teeth in the interviewees, regardless of whether they received either SC or IR. The IR-interviewees generally spent longer periods of time in these phases compared to SC-interviewees. In some but not all participants, these phases, and the feelings associated with them, had negative effects on their psychosocial wellbeing. This finding emphasises the importance of identifying the individuals at risk of experiencing negative effects on their psychosocial wellbeing; they may benefit from temporary interventions, or if possible an earlier start to treatment, to alleviate their dental anxiety. The IR-interviewees also risked experiencing these feelings during phase 5 because of breakage of the temporary prosthesis used from completion of pre-prosthetic orthodontics to IR.

Furthermore, it was evident that the IR-interviewees experienced increased feelings of different dental appearance due to missing teeth during active treatment, which is most likely because of the occurrence of space enlargement. Although, it is known that the use of pontics during treatment enhance the dental aesthetics aiming to alleviate dental anxiety [44], it is unknown whether the interviewees in this study were offered any pontics during their orthodontic treatments.

The interviewees, including both SC and IR recipients, explained that they had to spend their time differently from their untreated peers, as was explored in the ‘different due to treatment appointments’ sub-category. Orthodontic treatment is considered burdensome and time-consuming, and it requires taking time away from school or work and reducing the possibilities to engage in social activities [45, 46]. Time spent at the orthodontists has minimal impact on school performance according to parents and patients [46], which is in agreement with Hancock et al. [47, 48] who state that authorised absences from school have much less of an impact on academic performance than do unauthorised absences. Even if time spent at the orthodontist does not affect the school performance, it does contribute to the overall experience of being different.

In line with the grounded theory approach, this article provides an interpretative understanding of an interviewee’s perceived reality rather than seeking the ‘truth’ [49, 50]. As proposed by Charmaz [51], it is important to recognise that researchers play an active role in the research process. The pre-understandings, or the pre-conceptions of the researcher create a risk of imposing biases if they are not acknowledged [52]. The first author of this study (CH), as well as EJ and RL, has a background as a specialist in orthodontics and is familiar with the research area of MMLIs. Constant discussions were carried out between the authors throughout the process to ensure that the pre-understanding of CH did not influence the collection or analysis of the data in this study.

### Study strengths

Conducting a qualitative study, based on interviews, has enabled the investigation of how the treatment to address MMLIs is experienced by patients. The results highlight the fact that the interviewees considered treatment as starting at the time of diagnosis, and that experiences of being different is of importance during orthodontic treatment.

Another strength of this study is that it gave patients an opportunity to make their voices heard with regard to their treatments. It made different perspectives visible and resulted in the identification of phases and feelings about treatments that were otherwise at risk of being lost.

### Study limitations

The pre-understanding of the interviewer and author, CH, must be considered as a limitation of this study, even though these were actively dealt with during the different study processes to ensure that they made minimal impact on the study.

The lack of generalisability when conducting a qualitative study may be seen as a limitation. However, generalisation is not the purpose of qualitative research, which is, rather, to gain an in-depth understanding about a phenomenon based on research describing participants’ personal experiences.

## Conclusion

Patients with MMLIs consider their treatment to start at the time of diagnosis. They experience being different irrespective of their type of treatment, whether this is orthodontic SC or IR. However, each participant’s experiences of being different varies in timing and causes depending on the treatment received.
